# Potential Regulatory Effects of miR-182-3p in Osteosarcoma via Targeting EBF2

**DOI:** 10.1155/2019/4897905

**Published:** 2019-03-12

**Authors:** Gaoyang Chen, Wenqing Yu, Zhaoyan Li, Qingyu Wang, Qiwei Yang, Zhenwu Du, Guizhen Zhang, Yang Song

**Affiliations:** ^1^Department of Orthopedics, the Second Hospital of Jilin University, Ziqiang Street 218, Changchun, Jilin 130041, China; ^2^Research Centre of the Second Hospital of Jilin University, Ziqiang Street 218, Changchun, Jilin 130041, China; ^3^The Engineering Research Centre of Molecular Diagnosis and Cell Treatment for Metabolic Bone Diseases of Jilin Province, Ziqiang Street 218, Changchun, Jilin 130041, China; ^4^Hematology Department of Yantai Affiliated Hospital of Binzhou Medical University, Jinbu Street No. 717, Yantai, Shandong 264100, China

## Abstract

Osteosarcoma (OS) is one of the most common primary malignant bone tumors in adolescents with a high mortality rate. MicroRNA (miRNA) is a kind of noncoding RNAs and has been proved to participate in many physiological processes. Many miRNAs have been reported to act as function regulators in OS. In our study, the miRNA and gene expression profiles of OS were downloaded from GEO Datasets and the differential expression analysis was performed using GEO2R. 58 up- and 126 downregulated miRNAs were found. In the three OS gene profiles, 125 up- and 27 downregulated genes were found to be differentially expressed in at least two profiles. The miRNA-mRNA networks were constructed to predict the potential target genes of 10 most up- and downregulated miRNA. Venn analysis was used to detect the coexpressed differentially expressed genes (DEGs). EBF2, one of the upregulated DEGs, was also a potential target gene of miR-182-3p. Knockdown and overexpression of miR-182-3p resulted in overexpression and downexpression of EBF2 separately. Luciferase reporter gene experiment further verified the binding site of miR-182-3p and EBF2. CCK8 assay showed that miR-182-3p knockdown can further enhance the proliferation activity of OS cells, while overexpressing miR-182-3p can inhibit the proliferation activity of OS cells. Our research indicated that downexpression of miR-182-3p in OS cells results in overexpression of EBF2 and promotes the progression of OS.

## 1. Introduction

Osteosarcoma (OS), occurring primarily in children and adolescents, is the most common skeletal tumor disease [1]. It accounts for 3–5% of newly diagnosed cancers of children and with an observed initial peak between the age of 10-14 years [2, 3]. OS is with a high mortality rate resulting from its complex pathological processes and metabasis in primary stage [4–6]. The five-year survival rate of OS cases has improved to 60%–75% since the introduction of chemotherapy. However, the adverse effects accompanied by chemotherapy increased the sense of urgency to find new biological markers or specific molecular targeted therapeutic approaches in order to improve the clinical outcomes in OS patients [7].

MicroRNA (miRNA) is a kind of evolutionarily conserved small noncoding RNAs (ncRNAs) with a length of 22–24 nucleotides and has been reported to play crucial roles in the pathological process of disease and considered as new cancer biomarkers [8, 9]. It has been proved to control many physiological processes such as proliferation, differentiation, development, and apoptosis of cells via regulating hub gene expression [9–11]. To data, many studies have shown that the differential expression of miRNA might contribute to the initiation and progression of OS [12]. The miRNA miR-1284 was reported to function as a new regulator to suppress proliferation and migration of osteosarcoma cell by targeting HMGB1 [13]. Huang et al. showed the tumor suppresses the function of miR-124 by targeting Snail2 in OS cells, which indicated miR-124 might play critical roles in the progression of OS [14]. Other miRNAs (such as miR-143, miR-382, and miR-223) have also been demonstrated to deregulate expression in OS and proved to have potential use for OS prognosis, diagnosis, and therapeutic studies [12].

However, the role of miRNAs in OS still needs further research and validation. The rapid development of bioinformatics technology has brought us great convenience to search for molecular biological information of diseases. In this study, we coanalyzed one miRNA expression profile and three mRNA expression profile in order to find new OS-related miRNAs and further investigated their potential role in OS via regulating their target genes. 

## 2. Materials and Methods

### 2.1. Differential Expression Analysis of miRNA and Gene Profiles of OS

The miRNA and gene expression profiles of OS were searched from the Gene Expression Omnibus (GEO) database of the National Center of Biotechnology Information (NCBI, http://www.ncbi.nlm.nih.gov/geo/) [15]. Then these profiles were analyzed via GEO2R (https://www.ncbi.nlm.nih.gov/geo/geo2r/), an interactive online tool, which was widely applied to analyze differential expression of profiles. The adjusted* p *< 0.05 and |fold change (FC)| ≥ 2 were set as the criterions.

### 2.2. Coanalysis of Gene Profiles

The significantly differentially expressed genes (DEGs) of gene expression profiles were analyzed via Functional Enrichment analysis tool (FUNRICH) [16]. The coexpressed DEGs in these profiles were then acquired by using Venn analysis. The up- and downregulated DEGs were analyzed separately. DEGs which were found differentially expressed in at least two profiles were thought to be as coexpressed DEGs. Then these DEGs were analyzed using OmicsBean online tool in order to better understand their pathway enrichment.

### 2.3. Construction of miRNA-mRNA Networks

In order to predict the potential target genes of significantly differentially expressed miRNAs, miRNA-mRNA networks were constructed. The potential target genes of ten most up- and downregulated miRNAs were predicted via TargetScan (www.targetscan.org) and miRDB (www.mirdb.org). Then their interaction relationships were established by using Cytoscape application.

### 2.4. Cell Culture and Quantitative Real-Time PCR Analysis

Four human osteosarcoma cell lines, MG63, U2OS, 143B, and HOS, and the human mesenchymal stem cell line hMSCs were obtained from the American Type Culture Collection (ATCC). These cell lines were cultured in DMEM mixed with 10% fetal bovine serum and 1% antibiotics (streptomycin and penicillin). The culture medium was replaced every 2-3 days.

Total RNAs were extracted using TRIzol (Invitrogen) following the manufacturer's introductions. The cDNAs of miRNA were synthesized from 2 *μ*g of total RNA with an All-in-one™ miRNA First-Strand cDNA Synthesis (GeneCopoeia) Kit. While the cDNAs of mRNA were synthesized by reverse transcription from total RNA using Oligo(dT) priming method (PrimeScriptTMRT Reagent Kit; TaKaRa, Japan). Then quantitative real-time PCR was performed using SYBR Green qPCR Master Mix (Thermo Fisher Scientific). The expression of miRNA was normalized relative to U6 and the expression of mRNA was normalized to the GAPDH. The 2^−△△Ct^ method was used to calculate their relative expression.

### 2.5. Functional Verification of miRNA in OS Cell Line

The miRNA inhibitor and mimics of miR-182-3p were synthesized by Genepharma (Suzhou, China). The miRNA inhibitor was used to knockdown miR-182-3p and the mimics were used to overexpress miR-182-3p in MG63 cells, which was transfected using GoldenTran-R (Golden Trans Technology, Changchun, China). Forty-eight hours after transfection, the expression levels of miR-182-3p and target gene were then detected using quantitative real-time PCR.

### 2.6. Luciferase Reporter Gene Assay

The 3′-UTR of EBF2 containing the putative binding site (wide and mutated type) of miR-182-3p was inserted between the restrictive sites Xho I and Bgl II of pGL6-miR and validated by sequencing. The 293T cells were transfected with wild-type or mutated reporter vectors via GoldenTran-DR (Golden Trans Technology, Changchun, China), while the mimics and negative control were transfected into 293T cells via GoldenTran-R. Firefly luciferase activities were consecutively measured according to the dual-luciferase assay manual (Beyotime).

### 2.7. Cell Counting Kit-8 (CCK-8) Assay

The effect of miR-182-3p on the osteosarcoma cell proliferation activity was evaluated by CCK-8 assay. The MG63 cells were seeded into 96-well plate at a density of 3 × 10^3^ cells/well and cultured in a humidified incubator (37°C, 5% CO2) adding 10 *μ*L CCK8 assay solution into each well after transfection of miR-182-3p inhibitor or mimics at 1, 2, 3, 4, and 5 days and then incubated for 2 h. The optical density (OD) of each well was measured using a Multiskan Spectrum (Thermo Fisher Scientific, USA) at a wavelength of 450 nm.

### 2.8. Data Analysis

A value of* p *< 0.05 indicated that the difference was statistically significant. |FC| ≥ 2 were regarded as significantly differentially expressed criterion of miRNA and genes. Bar graphs were constructed by GraphPad Prism 7.0. 

## 3. Results

### 3.1. Differentially Expressed miRNA and Genes

**Figure 1:**
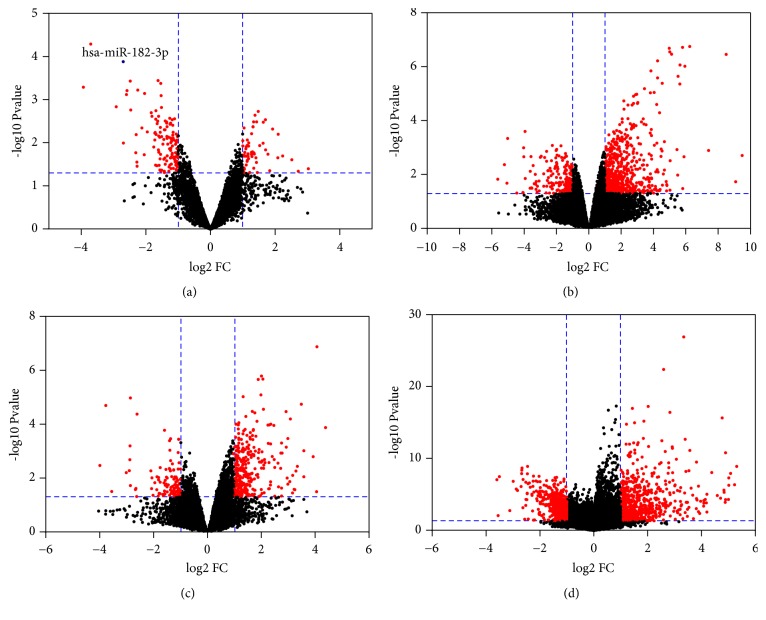
The volcano plots were constructed using fold-change values and P values, and the differentially expressed miRNAs or genes were signed in red. (a) Volcano plot of miRNA profile GSE70367: the researched miRNA miR-182-3p was signed in blue; (b) volcano plot of gene profile GSE70415; (c) volcano plot of gene profile GSE32395; and (d) volcano plot of gene profile GSE42352.

One miRNA expression profile and three gene expression profiles were detected from GEO Datasets and all of these profiles set the hMSCs as the control group (Table 1). Based on the criterions (*p *< 0.05 and |FC| ≥ 2), 126 miRNAs were found to be differentially expressed in OS, including 58 up- and 126 downregulated ones (Figure 1(a)). Accordingly, 865 DEGs were achieved from the gene profile of GSE70415, including 648 up- and 217 downregulated ones (Figure 1(b)). 460 DEGs, including 353 up- and 107 downregulated DEGs, were obtained from the gene profile of GSE32395 (Figure 1(c)). And 1166 DEGs including 691 up- and 475 downregulated ones were obtained from the gene profile of GSE42352 (Figure 1(d)).

### 3.2. Coexpressed DEGs and Pathway Enrichment

**Figure 2:**
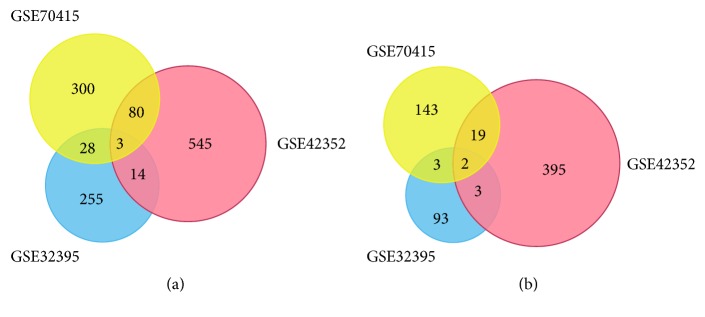
The Venn analysis of up- and downregulated DEGs. (a) 125 upregulated DEGs were found to be differentially expressed in at least two gene expression profiles. (b) 27 downregulated DEGs were found to be differentially expressed in at least two gene expression profiles.

The coexpressed DEGs of three gene expression profiles were obtained from the Venn Maps which were generated by FUNRICH. There were 125 up- and 27 downregulated DEGs found to be differentially expressed in at least two profiles (Figures 2(a) and 2(b)). In order to gain a better understanding of gene function, the pathway enrichment of these DEGs was analyzed using OmicsBean online tool. The top 5 enriched pathways of up- and downregulated DEGs were shown in Table 2.

### 3.3. The Regulation Networks of Differentially Expressed miRNA

**Figure 3:**
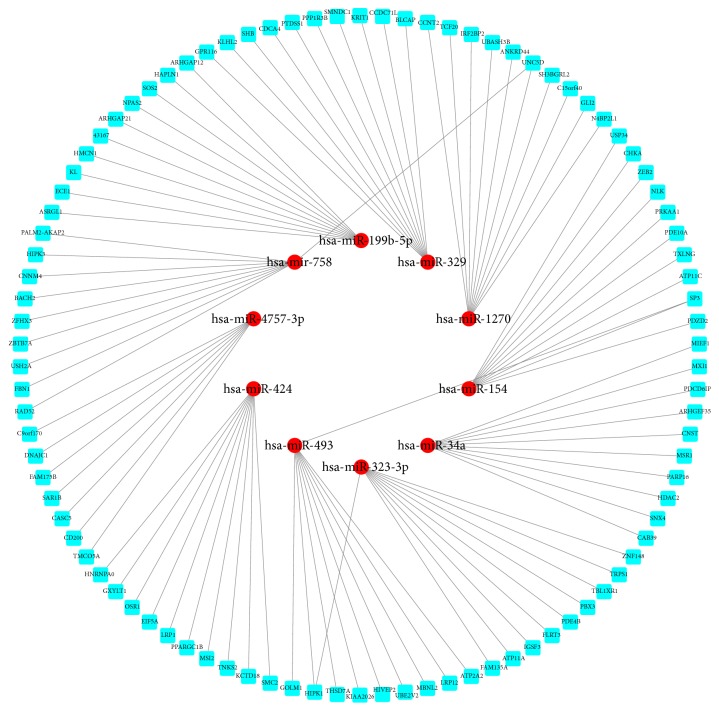
The regulating network of most 10 significantly upregulated miRNAs. The miRNAs were signed in red and their potential target genes were signed in light blue.

**Figure 4:**
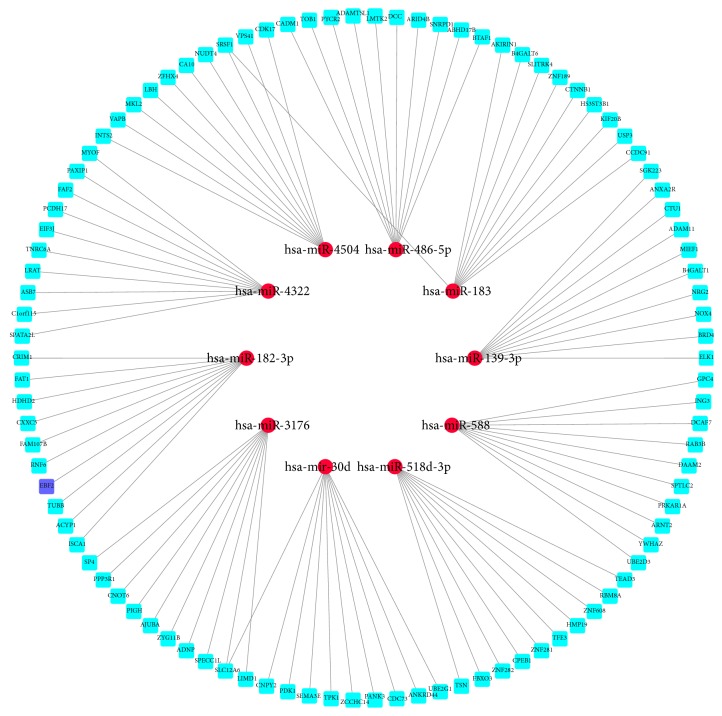
The regulating network of 10 most significantly downregulated miRNAs. The miRNAs were signed in red and their potential target genes were signed in light blue. The researched gene EBF2 was signed in deep blue.

The miRNA-mRNA regulation networks of ten most up- and downregulated miRNAs were generated separately via Cytoscape. These potential target mRNAs were predicted based on the TargetScan and miRDB. There were 97 mRNAs with high binding scores for 10 upregulated miRNAs and 100 mRNAs with high binding scores for 10 downregulated miRNAs were found. The regulation networks were shown in Figures 3 and 4. Among these mRNAs, we found the EBF2, which were predicted to be regulated by miR-182-3p and were also as coexpressed DEGs in above gene expression profiles.

### 3.4. Real-Time qPCR Validation of miR-183-3p and EBF2

**Figure 5:**
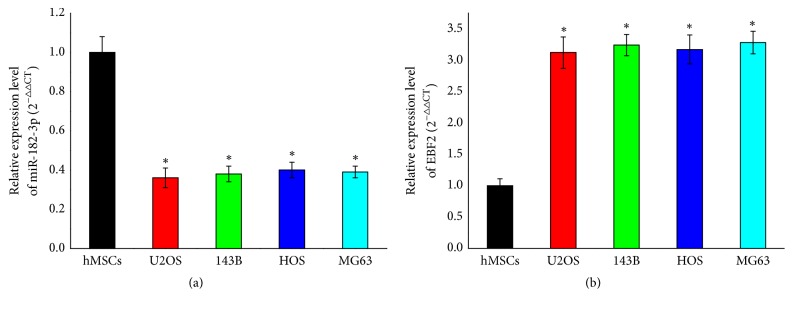
The expression level of (a) miR-182-3p and (b) EBF2 in four human osteosarcoma cell lines (U2OS, 143B, HOS, and MG63) and the human mesenchyma stem cell line (hMSCs) using real-time qPCR (n=3, ^*∗*^p<0.05).

The expression levels of miR-182-3p and EBF2 were validated in four OS cell lines via real-time qPCR. The results showed that miR-182-3p were with low expression levels in four OS cell lines compared to hMSCs, while EBF2 were found to be with high expression levels (Figure 5).

### 3.5. Functional Verification of miRNA

**Figure 6:**
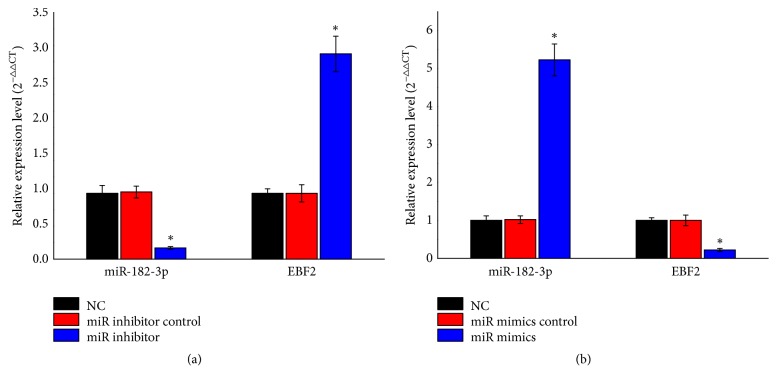
Functional verification of miR-182-3p. (a) Knockdown of miR-182-3p using miRNA inhibitor significantly increased the expression of EBF2 inMG63 cell lines. (b) Overexpression of miR-182-3p using miRNA mimics significantly suppressed the expression of EBF2 inMG63 cell lines (n=3, ^*∗*^p<0.05).

To analyze the role of miR-182-3p in EBF2 regulation in OS, we used miRNA inhibitor or mimics to knockdown or overexpress miR-182-3p expression level and then detected the expression level of EBF2 in MG63 cell line. The result showed that as the expression level of miR-182-3p declined, the expression level of EBF2 was found elevated (Figure 6(a)). As we overexpressed miR-182-3p via miRNA mimics, the expression level of EBF2 was found declined (Figure 6(b)).

### 3.6. Verification of miRNA Binding Sites

**Figure 7:**
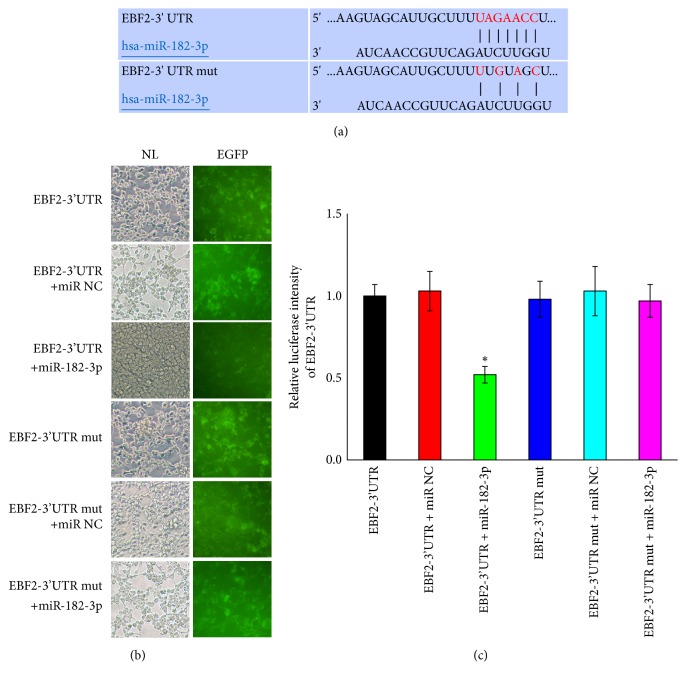
EBF2 is directly targeted by miR-182-3p. (a) Sequences of CDK6 3′-UTR and its mutant type. (b) 293T cells were cotransfected with an EGFP reporter plasmid (containing 3′UTR of EBF2 or its mutant type), either alone or in combination with a miR-182-3p mimic. EGFP levels were measured after 48 hours using fluorescence spectrophotometer. (c) Fluorescence intensity of all groups (n=3, ^*∗*^p<0.05).

Luciferase reporter assays were used to determine whether miR-182-3p can directly target the 3′UTR of EBF2. The alignment of miR-182-3p and the wild type and mutant type 3′UTR of EBF2 were constructed in Figure 7(a). The results showed that being cotransfected with mimics and wild type reporter plasmid significantly reduced the expression of EGFP in 293T cells. However, the EGFP expression level in 293T cells which cotransfected with mimics and mutant type reporter plasmid showed no significant change (Figures 7(b) and 7(c)).

### 3.7. The Effect of miR-182-3p on OS Cell Proliferation

**Figure 8:**
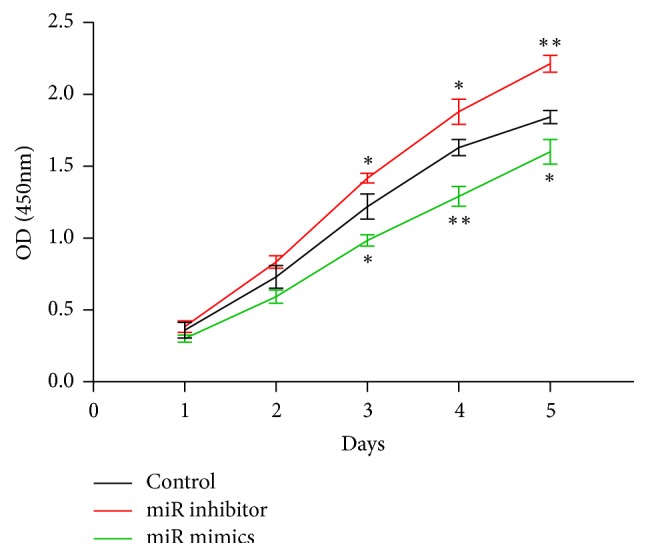
The miR-182-3p inhibitor enhanced the MG63 cell growth, while the miR-182-3p mimics inhibited the MG63 cell growth.

The proliferation of MG63 cells transfected by miR-182-3p inhibitor or mimics was determined by a CCK8 assay. The cell proliferation of MG63 cells was tested at 24 h intervals, for 5 days total. The proliferation of miR-182-3p inhibitor transfected MG63 cells has a significantly higher cell proliferation activity at 3, 4, and 5 days compared to the control group. However, MG63 cells transfected by miR-182-3p mimics showed a significantly lower cell proliferation activity at 3, 4, and 5 days (Figure 8).

## 4. Discussion

Many previous studies have shown a number of miRNAs participating in the progression process of OS [12]. In our study, we analyzed one miRNA expression profile of human OS cell lines, which was downloaded from GEO Datasets. We detected 58 upregulated and 126 downregulated miRNAs in OS cell lines. These differentially expressed miRNAs have the potential to act as molecular markers for diagnosis and treatment of OS. Lots of miRNAs have been proved to act as regulatory molecules via downregulated targeting genes in diseases [17]. Thus, we constructed regulation networks of 10 most significantly up- and downregulated miRNA. Their potential targeting genes were then clearly shown to us. Among these potential targeting genes, many have been reported to participate in the process of OS, such as cell adhesion molecule 1 (CADM1) [18], early B cell factor 2 (EBF2) [19], MAX interactor 1 (MXI1) [20], and nemo-like kinase (NLK) [21]. It indicated that these differentially expressed miRNAs may play critical regulating roles in the progression of OS.

We analyzed three gene expression profiles of OS and detected 125 up- and 27 downregulated coexpressed DEGs which were found to be differentially expressed in at least two gene expression profiles. For further understanding of the function of these coexpressed DEGs, pathway enrichment analysis was performed via OmicsBean. The results showed that upregulated coexpressed DEGs were enriched in pathways such as ECM-receptor interaction [22], focal adhesion [23], PI3K-Akt signaling pathway [24], platelet activation [25], and protein digestion and absorption [26]. The downregulated coexpressed DEGs were enriched in carbon metabolism [27], cell cycle [28], hepatitis B [29], and so on. These pathways have been proved to play crucial roles in OS progression.

There is an upregulated coexpressed DEG, early B cell factor 2 (EBF2) gene, also found as a potential target gene of miR-182-3p in the downregulated miRNA-mRNA regulation network. The protein encoded by this gene belongs to the COE (Collier/Olf/EBF) family and plays an important role in a variety of developmental processes. Previous studies in mouse suggested that this gene may be involved in the differentiation of osteoblasts [30]. Ana et al. identify relevant molecular targets in the pathogenesis of osteosarcoma via microarray analysis and found EBF2 was one of the most significantly overexpressed genes. They also found that high levels of EBF2 were associated with high OPG protein levels in osteosarcoma samples and knockdown of EBF2 result in stunted abrogation of OPG levels and increased sensitivity to tumor necrosis factor–related apoptosis-inducing ligand (TRAIL)–induced apoptosis [19]. Then study of Li et al. revealed that high level of EBF2 not only facilitated migration and invasion of osteosarcoma cells but also inhibited apoptosis [31].

We provided several lines of evidence implicating that miR-182-3p could regulate the expression of EBF2. First, the knockdown and overregulated miR-182-3p result in the overexpressed and downexpressed EBF2 separately. Second, the luciferase reporter gene experiment verified the binding site of miR-182-3p and EBF2. Thus, we proved that miR-182-3p could regulate EBF2 in OS cells. That is to say, the downexpression of miR-182-3p weakened its regulating function to EBF2 and resulted in the high expression level of EBF2. High level of EBF2, as the previous study showed, led to high OPG protein and decreased TRAIL-induced apoptosis, which further inhibited osteoclast genesis, tilted the balance of bone turnover, and favored aberrant bone formation [19]. The result of CCK-8 assay in our study also revealed that miR-182-3p knockdown can further enhance the proliferation activity of OS cells, while overexpressing miR-182-3p can inhibit the proliferation activity of OS cells.

In summary, we identified differentially expressed miRNAs and genes from one miRNA and three gene microarrays profiles. Further bioinformatics analysis found that the downexpressed miRNA miR-182-3p account for the EBF2 overexpression in OS cells, which was also proved by functional verification in OS cells. Our findings may provide new strategies in the diagnosis and therapy of OS.

## Figures and Tables

**Table 1:** The information of expression profiles.

Series	Probe types	OS cell lines	hMSCs
GSE70367	miRNA	5	1
GSE70415	mRNA	5	1
GSE32395	mRNA	6	1
GSE42352	mRNA	19	3

**Table 2:** Five most significant KEGG pathways of up- and downregulated co-DEGs separately.

Pathway terms	P value	Counts
Upregulated co-DEGs		
ECM-receptor interaction	2.16E-05	18
Focal adhesion	5.13E-04	27
PI3K-Akt signaling pathway	1.31E-03	30
Platelet activation	1.37E-03	16
Protein digestion and absorption	1.87E-03	12

Downregulated co-DEGs		
Small cell lung cancer	8.10E-20	2
Prostate cancer	8.05E-13	2
Carbon metabolism	2.93E-12	2
Cell cycle	1.19E-11	2
Hepatitis B	1.34E-11	2

## Data Availability

The data used to support the findings of this study are available from the corresponding author upon request.

## Abbreviations

OS: Osteosarcoma
miRNA: MicroRNA
ncRNAs: Noncoding RNAs
DEGs: Differentially expressed genes
GEO: Gene Expression Omnibus
FC: Fold change
FUNRICH: Functional enrichment analysis tool
OD: Optical density
CADM1: Cell adhesion molecule 1
EBF2: Early B cell factor 2
MXI1: MAX interactor 1
NLK: Nemo-like kinase
TRAIL: Tumor necrosis factor–related apoptosis-inducing ligand
CCK-8: Cell counting kit-8.
